# Supplementary material for: Unraveling the gut microbiota of Tibetan chickens: insights into highland adaptation and ecological advantages
Source: Microbiol Spectr. 2024 Sep 30;12(11):e00519-24. doi: 10.1128/spectrum.00519-24 (PMC11536995; doi:10.1128/spectrum.00519-24)
Supplement: Supplemental material — Tables S1 and S2; Fig. S1 to S4. [file spectrum.00519-24-s0001.docx]

**Supplementary Information**

**Unraveling the gut microbiota of Tibetan chickens: insights into highland adaptation and ecological advantages**

**Zeng, et al.**

**This PDF file includes:**

**Table S1-S2**

**Fig S1-S4**

**Table S1.** Statistics of metatranscriptome sequencing.

| **Sample ID** | **Raw reads** | **Clean reads** | **Q20 (%)** | **Q30 (%)** | **Effective reads** | **rRNA rate (%)** |
| --- | --- | --- | --- | --- | --- | --- |
| In-TC1 | 129,401,352 | 128,215,136 | 98.68 | 95.38 | 128,215,136 | 0.26 |
| In-TC2 | 135,336,286 | 133,489,090 | 98.89 | 96.21 | 133,489,090 | 0.05 |
| In-TC3 | 111,574,004 | 110,159,334 | 97.78 | 93.00 | 110,159,334 | 0.08 |
| In-TC4 | 74,727,306 | 73,489,072 | 98.77 | 95.85 | 73,489,072 | 0.06 |
| Ex-TC1 | 115,343,762 | 113,831,648 | 98.74 | 95.68 | 113,831,648 | 4.43 |
| Ex-TC2 | 111,031,764 | 109,990,992 | 98.87 | 96.00 | 109,990,992 | 4.12 |
| Ex-TC3 | 127,205,448 | 124,832,924 | 99.05 | 96.61 | 124,832,924 | 4.14 |
| Ex-TC4 | 101,317,112 | 100,333,040 | 99.17 | 97.02 | 100,333,040 | 5.13 |
| QY1 | 78,617,904 | 77,470,916 | 98.35 | 94.70 | 77,470,916 | 2.25 |
| QY2 | 97,399,160 | 96,462,322 | 98.90 | 96.10 | 96,462,322 | 7.32 |
| QY3 | 128,183,328 | 127,191,294 | 99.03 | 96.42 | 127,191,294 | 3.59 |
| QY4 | 103,345,466 | 101,480,036 | 99.23 | 97.11 | 101,480,036 | 6.65 |

**Table S2.** Statistics of assembly and gene recognition.

| **Sample ID** | **Contig num** | **Contig length (bp)** | **N50 (bp)** | **ORF num** | **Ave length (bp)** |
| --- | --- | --- | --- | --- | --- |
| In-TC1 | 108,613 | 102,602,751 | 948 | 156,532 | 624.69 |
| In-TC2 | 24,679 | 20,181,415 | 802 | 31,654 | 617.13 |
| In-TC3 | 134,134 | 124,810,777 | 918 | 197,199 | 597.97 |
| In-TC4 | 43,882 | 40,414,479 | 929 | 60,576 | 642.27 |
| Ex-TC1 | 26,594 | 20,768,573 | 751 | 29,121 | 415.38 |
| Ex-TC2 | 1,044 | 786,369 | 732 | 1,234 | 518.02 |
| Ex-TC3 | 18,406 | 14,242,890 | 751 | 24,181 | 537.86 |
| Ex-TC4 | 1,023 | 750,109 | 704 | 1,228 | 541.01 |
| QY1 | 6,807 | 5,218,682 | 739 | 9,096 | 525.42 |
| QY2 | 8,850 | 8,273,347 | 945 | 12,488 | 611.70 |
| QY3 | 9,859 | 8,105,866 | 804 | 13,306 | 558.95 |
| QY4 | 3,201 | 2,538,041 | 757 | 4,059 | 527.18 |

**Fig S1.** Differences in the inter-individual variations of active microbiome among different populations based on the Bray-Curtis distance.


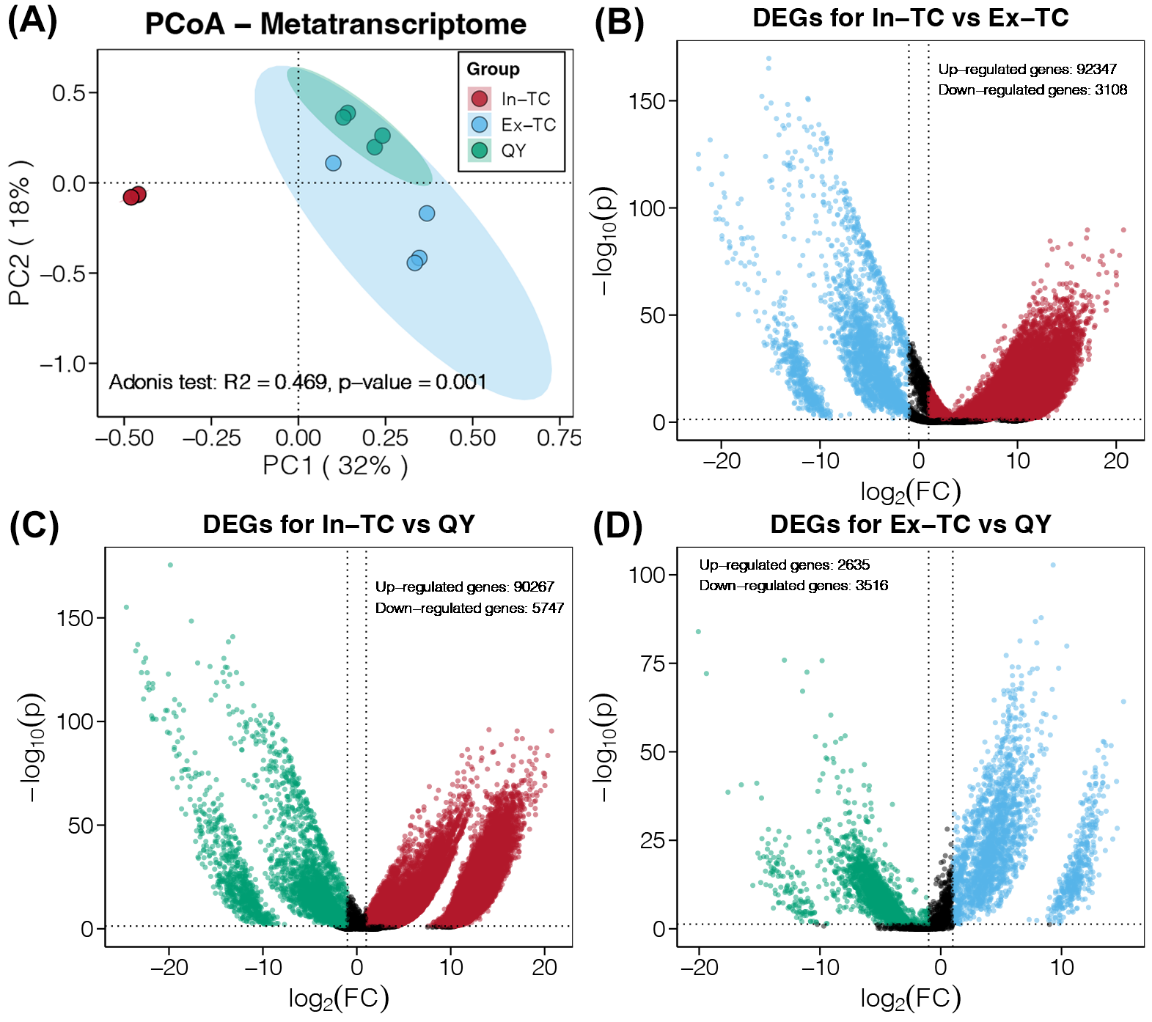


**Fig S2.** Identification of DEGs in the cecal microbiome of chicken populations. (A) PCoA illustrating the differences in gene expression profiles of the cecal microbiome among the three studied chicken populations. Volcano plots were used to identify the DEGs in cecal microbiome in the comparisons of In-TC vs. Ex-TC (B), In-TC vs. QY (C), and Ex-TC vs. QY (D), respectively.


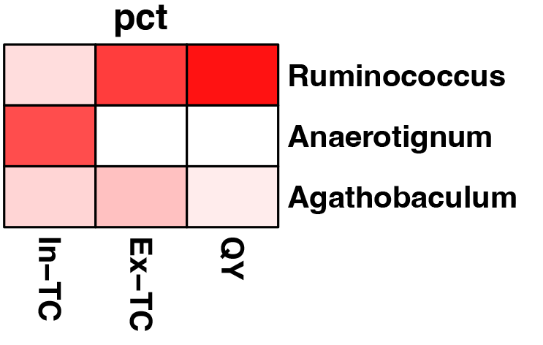


**Fig S3.** Host composition of the *pct* gene in cecal microbiome of chickens.


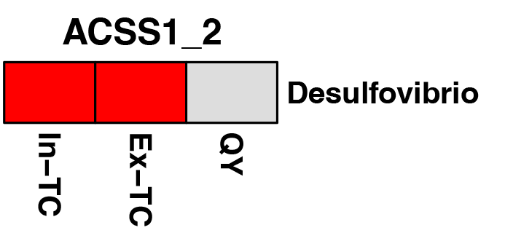


**Fig S4.** Host composition of the *ACSS1_2* gene in cecal microbiome of chickens.
